# Dietary Pattern, Hypertension and Cognitive Function in an Older Population: 10-Year Longitudinal Survey

**DOI:** 10.3389/fpubh.2018.00201

**Published:** 2018-07-20

**Authors:** Xiaoyue Xu, Deborah Parker, Zumin Shi, Julie Byles, John Hall, Louise Hickman

**Affiliations:** ^1^Faculty of Health, University of Technology, Sydney, NSW, Australia; ^2^Priority Research Centre for Generational, Health and Ageing, School of Medicine and Public Health, Hunter Medical Research Institute, University of Newcastle, Newcastle, NSW, Australia; ^3^Human Nutrition Department, Qatar University, Doha, Qatar; ^4^School of Medicine, University of Adelaide, Adelaide, SA, Australia; ^5^School of Public Health and Community Medicine, University of New South Wales, Sydney, NSW, Australia

**Keywords:** dietary pattern, cognitive functioning, hypertension, longitudinal data analysis, older people

## Abstract

**Background:** There is a paucity of studies that have explored the association between dietary pattern and cognitive function, and whether there is an interaction between dietary pattern and hypertension in relation to older people's cognitive functioning.

**Methods:** We analyzed data from the China Health and Nutrition (CHNS) survey. Dietary data have been collected since 1991, and cognitive function interview data were collected between 1997 and 2006. We analyzed ten years of data, including 4,847 participants with 10,658 observations (aged ≥55 years). Exploratory factor analysis was used to identify dietary patterns. Cognitive function measures include cognitive global scores and verbal memory scores. Linear mixed models were used to investigate the association between dietary patterns, hypertension and cognitive function.

**Results:** Three dietary patterns were identified by factor analysis, named “Traditional Chinese,” “Protein-rich,” and “Starch-rich” dietary pattern. A Protein-rich dietary pattern (high intake of milk, eggs and soymilk) was significantly associated with higher cognitive global scores and verbal memory scores, while the starch-rich dietary pattern (high intake of salted vegetable and legumes) was significantly associated with lower cognitive global and verbal memory scores. In addition, we found that participants with hypertension were independently associated with significant low cognitive function.

**Conclusion:** The study reinforces the importance of diet in preventing cognitive decline among the older population. Identification of older populations who had hypertension should be targeted in intervention studies to maintain their cognitive health.

## Introduction

China is aging rapidly. It is predicted that the proportion of population aged 60 years and over will increase from 14.3% in 2012 to 25% in 2035, and further increase to 40% in 2040 (1). This change in age structure has an impact on the increasing incidence of age-associated diseases or conditions, such as cardiovascular disease, arthritis, type 2 diabetes, hypertension, cognitive decline, and dementia (2).

Age remains the strongest risk for cognitive decline and dementia (3, 4). Globally, an estimated 47 million people were living with dementia in 2015 (5), with the incidence of dementia doubling with every 5.9 year increase in age, from 3.1/1,000 person yeas at age 60–64, to 175/1,000 person years at age 95 or above (6). In China, the number of dementia cases was 9.19 million (20% of the worldwide total) in 2015, and is predicted to increase to 30 million in 2050 (7, 8). People with Mild Cognitive Impairment (MCI) are more likely to develop dementia. A recent epidemiological study in China showed that 20.1% of people aged 60 or above had MCI, which implies the risk of high prevalence of dementia (9).

The high incidence and prevalence of cognitive impairment and dementia are leading chronic disease contributors to disability (10). People with cognitive impairment and dementia are at greater risk of disease comorbidities, hospital admission and subsequent mortality than their cognitively normal counterparts (10). They also have high needs for both social and health care, which contributes to an increasing economic burden (6). The total estimated worldwide cost of dementia was US $604 billion in 2010 (6).

Major modifiable risk factors for developing cognitive impairment and dementia including smoking, physical activity (8), obesity, and social engagement (5). Diet has been reported as a strong factor associated with chronic diseases among the older Chinese population (11–14). There is growing interest in the association between diet, cognitive functioning, as well as dementia (15–17). The link between single food (nutrient) and cognitive function has been proposed, with the mechanism of lowering oxidative stress, reducing inflammation, preventing vascular comorbidity, and protection against cerebrovascular diseases (18). For example, epidemiological evidence suggests the benefits of fish consumption, monounsaturated fatty acids, polyunsaturated fatty acids, and cognitive activity, in reducing the risk of cognitive decline and dementia (18). Dietary pattern has emerged in nutrition epidemiology studies to examine the impact of diet on health outcomes, as they illustrate the combined effects of diet intake (19). A Mediterranean (Med) diet, which includes high consumption of olive oil, legumes, unrefined cereals, fruits, and vegetables, has generated positive results suggesting its beneficial role for cognitive health (18). A systematic review by Rest et al. (20) found an inverse association between Med dietary pattern and cognitive functioning among four of six cross-sectional studies and six of twelve longitudinal studies. A systematic review from Aridi et al. (21) found Med diet is associated with better cognitive function, and might also decrease the risk of developing Alzheimer's disease. However, epidemiologic evidence of the association between dietary pattern (rather than Med dietary pattern) and cognitive function is mixed (22, 23). There are limited data on diet and cognition in China, mostly due to the complexity of collecting comprehensive data on cognitive functioning.

In addition, cardiovascular risk factors, such as diabetes, hypertension, and mid-life obesity, were associated with an increased risk of cognitive decline and dementia (5, 24). Our research team has previously identified an association between dietary pattern and hypertension (12). It may follow the interaction between dietary pattern and hypertension impacts on people's cognitive health.

Therefore, the purpose of this present study was to evaluate the association between dietary pattern and cognitive function, and to provide the evidence for clinical intervention and public health dietary guidelines in preventing cognitive health among older people. The specific aims were (1) to examine the association between dietary pattern and cognitive function, and identify individual foods, which are associated with cognitive function; and (2) to test whether there is an interaction between dietary pattern and hypertension in relation to cognitive function.

## Materials and methods

### China and health nutrition survey (CHNS)

The CHNS is an ongoing open cohort longitudinal survey of nine waves (1989–2011). The survey used a multistage random-cluster sampling process to select samples from nine provinces. Details of CHNS sampling is described in our previous studies (11, 13, 14, 25, 26). Briefly, the survey used a multistage random-cluster sampling process to select about 4,400 households with over 19,000 individuals from 9 provinces. Counties in the provinces were stratified by income levels, and a weighted sampling scheme was used to randomly select four counties in each province. Two cities (a provincial capital/or a big city and a lower-income city) were selected. Two urban neighborhoods, two suburban neighborhoods within cities, one county capital town and three villages within counties were randomly selected. Finally, twenty households were randomly selected within each neighborhood. As CHNS is an open cohort study, and the specific number of communities, household and individuals for each wave are presented in the CHNS website (27). Dietary data have been collected since 1991. Cognitive function data have been collected through interview between 1997 and 2006. In the analysis sample, 68% of participants attended the survey at least two times over the 10-year period.

### Dietary assessment and food group

Dietary assessment is based on each participant's 24 h-recall, with information collected over three consecutive days. The three consecutive days during which detailed food consumption data have been collected were randomly allocated from Monday to Sunday. Over 99% of the participants provided all of the 3 days dietary data. Details of the dietary data collection are described elsewhere (14, 25).

The food groups included were based on a food system developed specifically for the CHNS and the Chinese Food Composition Table, which has been applied in our previous studies (11, 28). Food categories were collapsed into 35 food groups based on the similar nutrient profiles or culinary use, and average food intake for individuals calculated for each wave, as Liang (Chinese ounce, 1 Liang = 50 g) (28).

### Hypertension

After at least a 10 min rest, trained examiners measured the blood pressure on the right arm in the sitting position using a mercury sphygmomanometer according to a standard protocol (12). Hypertension was defined by combining a systolic blood pressure (SBP) ≥ 140 mmHg and/or diastolic blood pressure (DBP) ≥ 90 mmHg or if the person was taking anti-hypertensive medication (12).

### Cognitive function

The CHNS adopted the cognitive screening items from part of the Telephone Interview for Cognitive Status-modified, which has been used in the Chinese population (15). The cognitive screening was administered among CHNS participants aged 55 years or over. The cognitive screening included immediate and delay recall of a 10-word list, counting backward from 20, serial 7 subtraction, and orientation (15). The outcomes of interest in the present study were repeated measures of cognitive global scores and include (1) immediate and delayed recall of a 10-word list (ten points each), counting backwards (two points), and serial 7's (five points); and (2) verbal memory scores which combined immediate and delayed 10-word recall (ten points each). High scores reflect better cognitive function.

### Covariates

Socio-demographic factors included in the study were age, gender, marital status (married and others), work status (Yes/No), education (low: illiteracy and primary school; medium: junior middle school; and high: high middle school or higher), and urbanization levels (low, medium, and high). Urbanization is defined by a multidimensional twelve-component urbanization index to capture population density and physical, social, cultural, and economic environments (7).

Health behavior factors included smoking, drinking and physical activity levels. Smokers were identified as people who smoke at least one cigarette per day, based on the question “how many cigarettes do you smoke per day?” Alcohol consumption was allocated to two categories (Yes/No), with the question “last year, did you drink beer or any other alcoholic beverage?” We calculated Metabolic Equivalent of Task (MET) to identify physical activity level based on the Compendium of Physical Activities. The details have been described in our previous studies (12–14). We also included doctor-diagnosed diabetes as a covariate given the strong link between diabetes and vascular dementia.

### Statistical analysis

**Figure 1:**
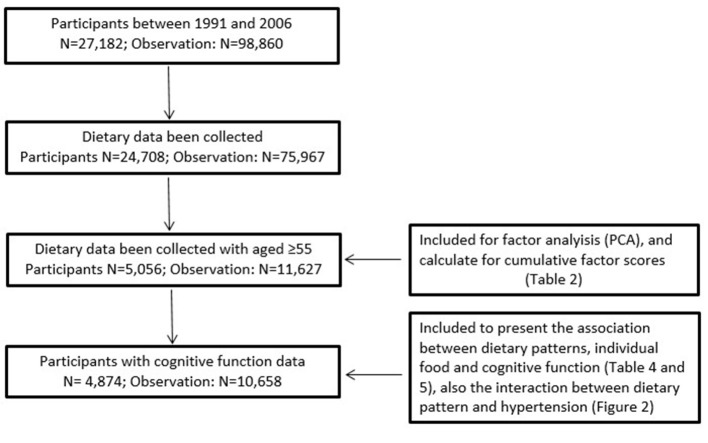
The number of participants in the analysis.

Study participants in each analysis are described in Figure 1. Dietary pattern across six waves (1991, 1993, 1997, 2000, 2004, and 2006) were identified by factor analysis, using the standard principal component analysis method (13, 14). The total participants aged 55 years or above, who also have dietary data were included in the factor analysis (*N* = 5,056, with 11,627 observations). The number of dietary patterns was identified based on the eigenvalue (>1), scree plot, factor interpretability, and the variance explained (>5%). Varimax rotation was applied to improve the interpretability of the factors and minimize the correlation between them. Factor loadings are equivalent to correlation between food items and factors. Higher loadings indicate a higher shared variance with the factor. Factor loadings of >|0.20| represent the foods that most strongly related to the identified factor.

At each wave, participants were assigned pattern-specific factor scores. Scores for each pattern were calculated as the sum of the products of the factor loading coefficients. We chose to use the cumulative mean score as it reflects long-term diet and may reduce dietary measurement error (28). Cumulative scores were added across seven waves (1991–2006), and a cumulative mean score was calculated for each factor.

Cumulative mean scores were divided into quartiles based on their distribution in each stratum, implying increased intake from quartile 1 (Q1) to quartile 4 (Q4). Mean and standard deviation across four quartiles were used to present the average global scores and verbal memory scores in each quartile of each dietary pattern.

Linear Mixed Models (LMMs) were used to evaluate associations between dietary patterns and cognitive function (both global scores and verbal memory scores). Model 1 adjusted for sociodemographic factors (age, gender, urbanization index, marital status, work status, and education level); model 2 adjusted for health behavior factors (BMI, alcohol drinking, and smoking status) and model 1; model 3 adjusted for survey years and model 2; and model 4 adjusted for hypertension, diabetes, and model 3.

LMMs were also used to examine the association between hypertension and both global scores and verbal memory scores. Model 1 adjusted for both dietary patterns; model 2 adjusted for sociodemographic factors and model 1; model 3 adjusted for health behavior factors and model 2; and Model 4 adjusted for diabetes and survey year and model 3.

### Ethics

Survey protocols, instruments, and the process for obtaining informed consent for CHNS were approved by the institutional review committees of the University of North Carolina at Chapel Hill and the National Institute of Nutrition and Food Safety, China Centre for Disease Control and Prevention. Also, this study has been approved by the University of Newcastle Ethics Committee, Australia (Approval Number: H-2013-0360).

## Results

### Sample characteristics

Table 1 shows the characteristics of study participants in 1997, 2000, 2004, and 2006. Significant differences were found between participants for different survey years in their age, physical activity levels, marital status, education level, work status, urbanization levels, alcohol drinking, BMI, smoking status hypertension, and cognitive global and verbal memory scores. No significant difference was found between different survey years and gender.

**Table 1:** Characteristics of study participants in 1997, 2000, 2004, and 2006 (*N* = 4,847 with 10,658 observations).

**Factors**	**1997**	**2000**	**2004**	**2006**	***P-*value**^‡^
**AGE**					
Median (IQR)	64 (59; 70)	64 (59; 71)	64 (59; 71)	64 (59; 71)	<0.001
**PHYSICAL ACTIVITY (MET)**					
Mean (SD)	88 (97)	77 (98)	76 (95)	73 (96)	<0.001
**GENDER**					
Men	1,006 (47.3%)	1,064 (47.8%)	1,418 (47.8%)	1,588 (47.5%)	0.93
Women	1,123 (52.8%)	1,160 (52.2%)	1,546 (52.2%)	1,753 (52.5%)	
**MARITAL STATUS**					
Married	1,530 (72.6%)	1,588 (74.7%)	2,286 (77.6%)	2,621 (78.6%)	<0.001
Other marital status^*^	577 (27.4%)	538 (25.3%)	660 (22.4%)	741 (21.4%)	
**EDUCATION LEVELS**					
Low	946 (49.6%)	781 (40.2%)	798 (27.0%)	1,017 (30.6%)	<0.001
Medium	621 (32.6%)	730 (37.7%)	1,295 (43.9%)	1,276 (38.4%)	
High	340 (17.8%)	426 (22.0%)	859 (29.1%)	1,034 (31.1%)	
**WORK STATUS**					
Yes	833 (39.3%)	826 (37.5%)	952 (32.2%)	1,046 (31.3%)	<0.001
No	1,286 (60.7%)	1,378 (62.5%)	2,005 (67.8%)	2,295 (68.7%)	
**URBANIZATION LEVELS**					
Low	782 (36.3%)	717 (32.2%)	955 (32.2%)	996 (29.8%)	<0.001
Medium	957 (45.0%)	797 (35.8%)	811 (27.4%)	998 (29.9%)	
High	390 (18.3%)	710 (31.9%)	1,198 (40.4%)	1,347 (40.3%)	
**SMOKING STATUS**					
Yes	574 (27.0%)	599 (26.9%)	779 (26.3%)	819 (24.5%)	<0.001
No	1,555 (73.0%)	1,625 (73.1%)	2,185 (73.7%)	2,522 (75.5%)	
**ALCOHOL DRINKING**					
Yes	641 (30.6%)	694 (31.9%)	863 (29.2%)	938 (28.1%)	<0.001
No	1,456 (69.4%)	1,484 (68.1%)	2,097 (70.8%)	2,403 (71.9%)	
**BMI**					
Underweight	249 (13.1%)	203 (9.4%)	244 (8.8%)	244 (7.8%)	<0.001
Normal	1,062 (56.0%)	1,214 (56.0%)	1,465 (52.5%)	1,656 (52.9%)	
Overweight	423 (22.3%)	563 (26.0%)	797 (28.6%)	934 (29.8%)	
Obese	164 (8.6%)	189 (8.7%)	284 (10.1%)	299 (9.5%)	
**HEALTH OUTCOMES**					
**Hypertension**					
No	1,081 (56.6%)	1,294 (59.3%)	1,705 (60.2%)	2,046 (64.2%)	<0.001
Yes	829 (43.4%)	887 (40.7%)	1,129 (39.8%)	1,139 (35.8%)	
**COGNITIVE GLOBAL SCORES**					
Median (IQR)	12 (7; 17)	13 (7; 18)	13 (8; 18)	12 (7; 17)	<0.001
**VERBAL MEMORY SCORES**					
Median (IQR)	9 (6; 12)	9 (6; 12)	9 (6; 12)	8 (5; 11)	<0.001

‡ Generalized Estimating Equation was used to examine the association between survey year and gender, marital status, work status, smoking status, alcohol drinking and hypertension. Mixed linear regression as used to examine the association between survey year and age, physical activity, education levels, urbanization level, BMI, cognitive global scores and verbal memory scores.

* *Other marital status includes divorced, widowed, separated, and never married*.

### Dietary patterns

Dietary patterns were constructed using factor analysis, with three factors explaining 15.7% of the variance in food intake. Factor 1 (“Traditional Chinese” dietary pattern) was loaded heavily on rice, pork and fish, and inversely on wheat and whole grain. Factor 2 (“Protein-rich” dietary pattern) was characterized by high intake of milk, soy milk and eggs, and inversely on rice and fresh vegetables. Factor 3 (“Starch-rich” dietary pattern) was characterized by high intake of salted vegetables, legumes, whole grain and tubers (Table 2).

**Table 2:** Factor loadings of three dietary patterns.

	**Factor loadings**		
	**Traditional Chinese**	**Protein-rich**	**Starch-rich**
Rice	0.72	−0.37	
Fruit		0.47	
Salted vegetables			0.66
Legume			0.55
Whole grain	−0.42		0.54
Tubers			0.47
Soy milk		0.43	
Milk		0.42	
Deep fired product		0.40	
Eggs		0.39	
Pork	0.48		
Fish	0.42		
Fast food		0.39	
Cake		0.31	
Dry tofu	0.31		
Fungus		0.26	
Shrimp		0.25	
Beer		0.23	
Poultry	0.30	0.22	
Nuts		0.21	
Fresh vegetable	0.26	−0.21	
Offal	0.25		
Beef	0.25		
Wheat	−0.70		

Table 3 shows the food intake across quartiles of three dietary patterns. For the traditional Chinese dietary pattern, compared with Quartile 1 (Q1), Q4 had significantly higher intakes of rice, pork, fish, dry tofu, poultry, fresh vegetable, offal, and beef; and significantly lower intakes of whole grain and wheat (*p* for trend <0.001).

**Table 3:** Food intake across quartiles of three dietary patterns.

**Food items (Liang per day)**	**Intake of Traditional Chinese dietary pattern (Q)**								***p* for trend**
	**Q1**		**Q2**		**Q3**		**Q4**		
	**Mean**	**SD**	**Mean**	**SD**	**Mean**	**SD**	**Mean**	**SD**	
Rice	1.04	1.31	4.56	2.01	6.46	2.03	7.35	2.48	<0.001
Pork	0.50	0.53	0.84	0.67	1.22	0.77	1.73	0.96	<0.001
Fish	0.10	0.23	0.28	0.42	0.51	0.54	1.10	0.89	<0.001
Dry tofu	0.04	0.13	0.13	0.22	0.23	0.32	0.33	0.39	<0.001
Poultry	0.05	0.15	0.10	0.25	0.18	0.29	0.48	0.60	<0.001
Fresh vegetables	5.00	2.06	5.00	2.16	5.82	2.25	6.50	2.28	<0.001
Offal	0.01	0.06	0.03	0.13	0.06	0.13	0.14	0.28	<0.001
Beef	0.02	0.09	0.05	0.15	0.10	0.20	0.19	0.32	<0.001
Whole grain	1.16	1.39	0.33	0.68	0.08	0.28	0.04	0.15	<0.001
Wheat	7.23	3.30	2.59	1.65	1.23	1.01	0.90	0.82	<0.001
**INTAKE OF PROTEIN-RICH DIETARY PATTERN (Q)**									
Fruit	0.05	0.18	0.13	0.35	0.33	0.60	1.22	1.72	<0.001
Soy milk	0.01	0.05	0.03	0.11	0.08	0.22	0.50	0.83	<0.001
Milk	0.001	0.22	0.005	0.04	0.05	0.20	0.80	1.26	<0.001
Deep fried products	0.01	0.03	0.02	0.07	0.09	0.19	0.34	0.51	<0.001
Eggs	0.17	0.20	0.28	0.26	0.54	0.43	0.99	0.83	<0.001
Fast food	0.003	0.02	0.01	0.08	0.05	0.13	0.19	0.44	<0.001
Cake	0.01	0.07	0.02	0.08	0.06	0.17	0.24	0.55	<0.001
Fungus	0.01	0.05	0.03	0.08	0.08	0.16	0.16	0.27	<0.001
Shrimp	0.003	0.02	0.01	0.05	0.03	0.12	0.14	0.48	<0.001
Beer	0.01	0.10	0.01	0.10	0.04	0.27	0.25	1.30	<0.001
Poultry	0.10	0.21	0.15	0.29	0.25	0.44	0.31	0.55	<0.001
Nuts	0.02	0.06	0.03	0.07	0.05	0.12	0.12	0.25	<0.001
Fresh vegetables	6.98	2.60	5.27	1.86	5.07	1.98	4.98	1.98	<0.001
Rice	7.81	2.34	4.45	2.82	4.03	2.80	3.10	2.33	<0.001
**INTAKE OF STARCH-RICH DIETARY PATTERN (Q)**									
Salted vegetables	0.04	0.08	0.11	0.14	0.19	0.22	0.49	0.62	<0.001
Legume	0.04	0.09	0.08	0.15	0.14	0.21	0.42	0.72	<0.001
Whole grain	0.16	0.29	0.24	0.49	0.37	0.70	0.85	1.49	<0.001
Tubers	0.36	0.43	0.54	0.56	0.81	0.82	1.53	1.67	<0.001

For the protein-rich dietary pattern, compared with Q1, Q4 had significantly higher intakes of fruit, soy milk, milk, deep fried products, eggs, fast food, cake, fungus, shrimp, beer, poultry, and nuts; and significantly lower intakes of vegetables and rice (*p* for trend <0.001). However, relative low intakes across four quartiles were found for milk, deep fried products and fast food. For starch-rich dietary pattern, compared with Q1, Q4 had significantly higher intakes of salted vegetables, legumes, whole grain and tubers (*p* for trend <0.001).

### Association between dietary patterns and cognitive function

Table 4 shows that there were significant positive association between protein dietary pattern and cognitive function for both global scores and verbal memory scores. The significant positive association was also found between traditional Chinese dietary pattern and cognitive global scores, but not for verbal memory scores. The significant negative associations were found between starch-rich dietary pattern and cognitive function for both global scores and verbal memory scores (*p* for trend <0.001).

**Table 4:** The association between dietary patterns and cognitive function^*^.

**Global scores**	**Quantiles of dietary pattern (Q)**							
	**Q1**	**Q2**		**Q3**		**Q4**		***P* for trend**
**Traditional Chinese**	**Ref**	**β**	**95% CI**	**β**	**95% CI**	**β**	**95% CI**	
Crude model	1	0.90	0.46; 1.33	0.34	−0.10; 0.78	2.79	2.36; 3.23	<0.001
Model 1	1	0.76	0.39; 1.13	0.39	0.01; 0.77	0.95	0.57; 1.33	<0.001
Model 2	1	1.13	0.73; 1.53	0.93	0.51; 1.35	1.39	0.98; 1.80	<0.001
Model 3	1	1.08	0.68; 1.47	0.81	0.39; 1.23	1.31	0.90; 1.72	<0.001
Model 4	1	1.10	0.70; 1.50	0.86	0.43; 1.28	1.32	0.90; 1.73	<0.001
**PROTEIN-RICH**								
Crude model	1	0.85	0.43; 1.28	2.35	1.93; 2.78	4.05	3.63; 4.48	<0.001
Model 1	1	0.86	0.49; 1.24	1.77	1.37; 2.16	2.47	2.02; 2.91	<0.001
Model 2	1	0.79	0.40; 1.19	1.73	1.31; 2.15	2.41	1.93; 2.89	<0.001
Model 3	1	0.73	0.33; 1.12	1.62	1.20; 2.04	2.27	1.79; 2.75	<0.001
Model 4	1	0.72	0.32; 1.12	1.66	1.24; 2.08	2.28	1.80; 2.76	<0.001
**STARCH-RICH**								
Crude model	1	−0.13	−0.57; 0.31	0.32	−0.12; 0.76	0.36	−0.08; 0.80	0.16
Model 1	1	−0.27	−0.63; 0.09	−0.32	−0.69; 0.04	−0.45	−0.83; −0.08	<0.001
Model 2	1	−0.27	−0.64; 0.10	−0.21	−0.58; 0.16	−0.47	−0.85; −0.09	<0.001
Model 3	1	−0.24	−0.61; 0.13	−0.14	−0.51; 0.23	−0.34	−0.73; 0.04	<0.001
Model 4	1	−0.20	−0.57; 0.18	−0.12	−0.50; 0.26	−0.31	−0.70; 0.08	0.001
**VERBAL MEMORY**								
**Traditional Chinese**								
Crude model	1	0.46	0.17; 0.76	−0.21	−0.51; 0.09	1.18	0.88; 1.47	<0.001
Model 1	1	0.75	0.46; 1.03	0.32	0.02; 0.61	0.54	0.24; 0.83	0.78
Model 2	1	0.76	0.47; 1.05	0.30	-0.01; 0.60	0.54	0.24; 0.84	0.78
Model 3	1	0.72	0.43; 1.01	0.18	-0.13; 0.48	0.46	0.16; 0.76	0.84
Model 4	1	0.73	0.44; 1.02	0.19	-0.11; 0.50	0.44	0.14; 0.74	0.77
**PROTEIN-RICH**								
Crude model	1	0.52	0.23; 0.82	1.38	1.09; 1.67	2.33	2.04; 2.61	<0.001
Model 1	1	0.53	0.25; 0.81	1.14	0.84; 1.43	1.56	1.23; 1.90	<0.001
Model 2	1	0.45	0.16; 0.74	1.05	0.75; 1.36	1.47	1.12; 1.82	<0.001
Model 3	1	0.39	0.10; 0.68	0.94	0.64; 1.25	1.33	0.98; 1.67	<0.001
Model 4	1	0.41	0.12; 0.70	0.99	0.69; 1.30	1.36	1.01; 1.71	<0.001
**STARCH-RICH**								
Crude model	1	−0.12	−0.42; 0.18	0.06	−0.24; 0.36	0.04	−0.26; 0.33	0.02
Model 1	1	−0.13	−0.40; 0.13	−0.30	−0.57; −0.04	−0.51	−0.78; −0.24	0.001
Model 2	1	−0.21	−0.48; 0.06	−0.31	−0.58; −0.04	−0.59	−0.87; −0.31	<0.001
Model 3	1	−0.18	−0.45; 0.09	−0.23	−0.50; 0.04	−0.46	−0.74; −0.18	<0.001
Model 4	1	−0.17	−0.44; 0.10	−0.22	−0.49; 0.05	−0.43	−0.71; −0.15	<0.001

* *Model 1 adjusted for age, gender, urbanization index, marital status, work status, and education levels; model 2 adjusted for BMI, alcohol drinking, smoking status, and model 1. Model 3 adjusted for survey year and model 2, model 4 adjusted for hypertension, diabetes, and model 3*.

In the crude model, compared with Q1, participants in the highest quartile of protein-rich dietary pattern had β (difference in mean) of 4.05 (95% CI: 3.63; 4.48) higher global scores, and 2.33 (95% CI: 2.04; 2.61) higher verbal memory scores. After adjustment for sociodemographic, health behaviors, survey years, diabetes and hypertension (model 4), participants in the highest quartile of protein-rich dietary pattern had β of 2.28 (95% CI: 1.80; 2.76) increases for global scores, and 1.36 (95% CI: 1.01; 1.71) increases for verbal memory scores.

In the crude model for traditional Chinese dietary pattern, compared with Q1, participants in the highest quartile had β of 2.79 (95% CI: 2.36; 3.23) higher global scores, and 1.18 (95% CI: 0.88; 1.47) higher verbal memory scores. After adjustment (model 4), participants in the highest quartile of traditional Chinese dietary pattern had of 1.32 (95% CI: 0.90; 1.73) increases for global scores (*p* for trend <0.001). No significant association was found between traditional Chinese dietary pattern and verbal memory scores after adjustment.

In the crude model for starch-rich dietary pattern, no significant association was found between this dietary pattern and global scores; while compared with Q1, participants in the highest quartile had β of 0.04 (95% CI: −0.26; 0.33) indicating higher verbal memory scores. In the adjusted model (model 4), compared with Q1, participants in the highest quartile had β of 0.31 (95% CI: −0.70; 0.08) lower global scores, and 0.43 (95% CI: −0.71; −0.15) lower verbal memory scores (*p* for trend <0.001).

**Figure 2:**
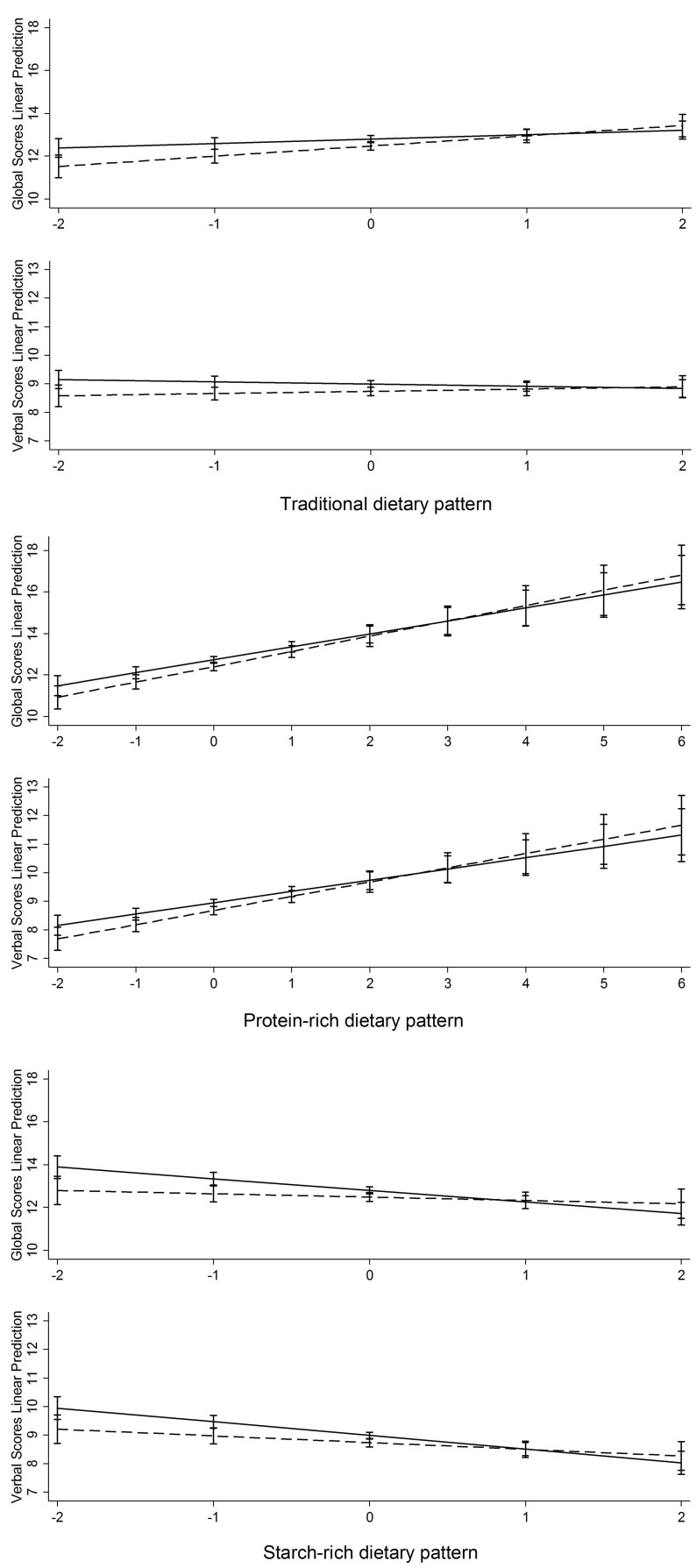
Interaction between dietary pattern and hypertension in relation to cognitive function. After adjusted for age, gender, urbanization index, marital status, work status, education levels, BMI, alcohol drinking, smoking status, diabetes and survey year. *P*-value for interaction: (1) Traditional Chinese dietary pattern: *p* = 0.07 for global cognitive scores, *p* = 0.15 for verbal memory scores; (2) Protein-rich dietary pattern: *p* = 0.43 for global cognitive scores, *p* = 0.32 for verbal memory scores; (3) Starch-rich dietary pattern: *p* = 0.05 for global cognitive scores, *p* = 0.10 for verbal memory scores.

Although no interactions (*p* > 0.05) were found between hypertension and three dietary patterns in relation to cognitive function (Figure 2), we found hypertension to be an independent factor which was significantly associated with both cognitive global and verbal memory scores. After adjustment for dietary pattern, sociodemographic, health behaviors, survey years, and diabetes, participants with hypertension had 0.32 (95% CI: −0.57; −0.07) lower cognitive global scores, and 0.26 (95% CI: −0.44; −0.07) lower verbal memory scores than those with no hypertension.

### Association between individual food intake and cognitive function

As the positive link was found between protein-rich dietary pattern and both cognitive global and verbal memory scores, and the negative link was found for starch-rich dietary pattern and both cognitive global and verbal memory scores, we further examined the association between each food component in these two dietary patterns and the cognitive function after adjusting for sociodemographic and health behavior factors. Due to the low intake of fruit, soy milk, milk, deep fired products, fast food, cake, fungus, shrimp, beer, poultry, nuts, salted vegetables, legumes, whole grain, we categorized consumption into two levels: non-consumers and consumers. Eggs and tubers were categorized into four quantiles, which indicates increased intake from Q1 to Q4.

Compared with fruit non-consumers, fruit consumers had 0.43 higher cognitive global scores (*p* < 0.001) and 0.03 higher verbal memory scores (*p* = 0.03); compared with soy milk non-consumers, soy milk consumers had 0.28 higher cognitive global scores (*p* = 0.01) and 0.30 higher verbal memory scores (*p* < 0.001); compared with milk non-consumers, milk consumers had 1.16 higher cognitive global scores (*p* < 0.001), and 0.77 higher verbal memory scores (*p* < 0.001); compared with Q1 for egg consumers, participants in the highest quantiles had 1.06 higher cognitive global scores (*p* < 0.001), and 0.75 for verbal memory scores (*p* = 0.002). Similar pictures were also found for fungus and poultry: compared with fungus and poultry non-consumers, fungus, and poultry consumers had significant higher cognitive global and verbal memory scores (*p* < 0.05) (Table 5A).

**Table 5:** Coefficients (95% CI) for cognitive function by quartiles of individual food intake for potential-rich and salted-vegetable dietary pattern^*^.

**Global scores**	**Quantiles of food components (Q)**				
	β **(95% CI)**				
**Food groups**	**Q1**	**Q2**	**Q3**	**Q4**	***P* for trend**
**(A) PROTEIN-RICH DIETARY PATTERN**					
Fruit	0	0.43 (0.14; 0.71)	–	–	<0.001
Soy milk	0	0.28 (-0.05; 0.62)	–	–	0.01
Milk	0	1.16 (0.76; 1.55)	–	–	<0.001
Deep fired product	0	−0.04 (-0.35; 0.27)	–	–	0.12
Eggs	0	0.32 (-0.06; 0.69)	0.88 (0.49; 1.26)	1.06 (0.66; 1.46)	0.001
Fast food	0	0.31 (-0.06; 0.69)	–	–	0.59
Cake	0	0.29 (-0.05; 0.64)	–	–	0.32
Fungus	0	0.53 (0.23; 0.83)	–	–	<0.001
Shrimp	0	0.37 (−0.01; 0.74)	–	–	0.05
Beer	0	0.71 (0.04; 1.38)	–	–	0.47
Poultry	0	0.67 (0.40; 0.94)	–	–	<0.001
Nuts	0	0.47 (0.17; 0.77)	–	–	0.002
**Verbal memory scores**					
Fruit	0	0.03 (−0.17; 0.24)	–	–	0.03
Soy milk	0	0.30 (0.05; 0.54)	–	–	<0.001
Milk	0	0.77 (0.48; 1.05)	–	–	<0.001
Deep fired product	0	0.15 (−0.07; 0.38)	–	–	0.61
Eggs	0	0.14 (−0.13; 0.42)	0.44 (0.16; 0.72)	0.75 (0.46; 1.04)	0.002
Fast food	0	0.07 (−0.20; 0.34)	–	–	0.73
Cake	0	0.15 (−0.10; 0.39)	–	–	0.25
Fungus	0	0.33 (0.12; 0.55)			<0.001
Shrimp	0	0.04 (−0.24; 0.31)	–	–	0.19
Beer	0	0.11 (−0.37; 0.59)	–	–	0.99
Poultry	0	0.19 (−0.01; 0.39)	–	–	0.03
Nuts	0	0.08 (−0.14; 0.30)	–	–	0.42
(B) STARCH-RICH DIETARY PATTERN*					
Salted vegetables	0	−0.24 (−0.51; 0.03)	–	–	0.01
Legumes	0	−0.37 (−0.63; −0.11)	–	–	<0.001
Whole grain	0	0.09 (−0.18; 0.36)	–	–	0.09
Tubers	0	0.14 (−0.23; 0.50)	−0.14 (−0.51; 0.24)	0.14 (−0.23; 0.51)	0.47
**Verbal memory scores**					
Salted vegetables	0	−0.44 (−0.63; −0.25)	–	–	<0.001
Legumes	0	−0.28 (−0.47; −0.10)	–	–	<0.001
Whole grain	0	1.11 (−0.09; 0.30)	–	–	0.17
Tubers	0	0.16 (−0.10; 0.43)	0.07 (−0.20; 0.35)	0.22 (−0.05; 0.49)	0.07

* *Adjusted for age, gender, urbanization index, marital status, work status, education levels, BMI, alcohol drinking, smoking status, survey year, hypertension and diabetes*.

For the starch-rich dietary pattern, compared with salted vegetables non-consumers, salted vegetable consumers had 0.24 lower cognitive global scores (*p* = 0.01) and 0.44 lower verbal memory scores (*p* < 0.001); Compared with legumes consumers, legumes non-consumers had 0.37 lower cognitive global scores (*p* < 0.001) and 0.28 lower verbal memory scores (*p* < 0.001). No significant associations were found between whole grain, tubers and cognition scores (Table 5B).

### Sensitivity analysis

As there was a chance that participants who had low cognitive function would not provide valid dietary information, we further conducted sensitivity analysis by excluding participants who had low cognitive global scores less than 5 (*N* = 1,398, 13% of total participants), and participants who had low verbal memory scores less than 3 (*N* = 835, 8% of total participants), respectively. In addition, as there was a potential association between heart disease and low cognitive functioning, and the association between diabetes and low cognitive functioning, we also excluded the participants who had heart disease (*N* = 294, 2.7% of total participants), and the participants who had diabetes (*N* = 358, 3.4% of total participants). However, the results were consistent with the full analysis.

## Discussion

The study identified three dietary patterns: a protein-rich dietary pattern was inversely associated with cognitive decline, while starch-rich dietary pattern was positively associated with cognitive decline for both cognitive global scores and verbal memory scores. No interaction was found between hypertension and dietary pattern impacting on cognitive health, but we found hypertension to be an independent factor which is significantly associated with cognitive function among older Chinese population.

Advancing age in China is associated with the high prevalence of cognitive decline and an increase in neurodegenerative disorders, such as dementia (8). Despite the proportionally large older population and massive demand for aged care, very little is known of effective measures for maintaining cognitive function among this population (29). A better understanding of the role of diet in maintaining cognitive function would benefit older people's quality of life, and further reduce the cost of dementia care among the older population.

Our study shows the significant decrease in verbal memory scores (include immediate and delayed recall words) among all participants between1997 and 2006. Although there is no consensus about the specific memory test that should be used to diagnose MCI or the prodromal phase of dementia, previous studies showed that delayed recall of the word list was the most successful discriminator to classify correctly 96% of normal subjects (30). An immediate and delayed recall of a 10-word list is a variation of Rey's Auditory Verbal Learning Test (31), and the study showed that delayed recall of words test is a sensitive measure for diagnosis of amnestic mild cognitive impairment and early dementia regardless of short or long-term recall (32).

Protein-rich dietary pattern was inversely associated with cognitive decline, with the milk, soy milk, egg and fungus the most influential diet factors in our results. Compared with people who did not consume milk, people who consumed milk had higher cognitive functioning. Relatively little attention has been paid to milk (33), and studies on the association between milk consumption and cognitive function were inconsistent. The results from US National Health and Nutrition Examination Survey show that compared with non-consumers, the people who consumed dairy foods had higher cognitive functioning. However, the significant association was only observed with cheese consumers, not for milk consumers (34). A recent meta-analysis among 10,940 participants shows that the higher consumption of milk was significantly associated with cognitive disorders. Compared with people with lowest level of milk consumption, people at the highest level of milk consumption had a decrease in cognitive disorders of 28% (35). The benefit of consuming milk in slowing the cognitive decline may be attributed to its protein, minerals, vitamins, and essential amino acids (35, 36). Although Chinese dietary guidelines present the benefit of consuming milk, our previous study showed that milk was consumed by an extremely small proportion of older people, with only 0.5% of them meeting the recommended intake of dairy (26). The small proportion of people consuming milk limits this study in drawing conclusions on the relationship between milk consumption and improvement in cognitive function.

Our results indicate that egg is the main dietary factor in the protein-rich dietary pattern for cognitive function among older people in China. Numerous studies have shown the association between high egg consumption and increased risk of coronary heart diseases, stroke (37) and incidence of Type 2 diabetes (38). However, eggs have been reported to be a highly bioavailable source of lutein and zeaxanthin, which may be important in cognitive function. Lutein can help maintain brain structure by lowering chronic oxidative stress; and both Lutein and Zeaxanthin are known to be potent anti-inflammatories as the brain is susceptible to damage due to chronic inflammation (39). A recent Finnish study that followed 2,497 adults for 22 years, found moderate egg intake was associated better with performance in neuropsychological tests, include executive functioning, and frontal lobe performance (40).

Our results show that compared with non-consumers, soy milk consumers had higher cognitive functioning, including both global and verbal memory scores. Previous studies suggested the beneficial role of soy consumption to health, for example, the link has been found between increased soy consumption and lower risk of incidence and prognosis of osteoporosis, coronary heart diseases (41), breast cancer (42), and diabetes (43). However, studies exploring the benefit of consuming soy products in cognitive functioning were inconsistent (44, 45). For example, 78 elderly individuals with mild cognitive deficits who were given 300 mg soy supplementation (Soybean-derived phosphatidylserine made from soybean lecithin) per day for six months showed a significant improvement in memory function compared with the placebo group (45); while the six months trials of 100mg/day soy isoflavone among 65 older people who were aged 60 years or over did not benefit people with Alzheimer's disease (46). No association between soy isoflavone and cognitive functioning was found among healthy, postmenopausal women during a 16 week intervention (47). The mechanism of these different effects need to be further explored.

Edible fungus (mushrooms and fungi) is often considered as an alternative medicine for treating age-related disease, include type 2 diabetes, cardiovascular disease and cancer (48). The medicinal effects attributed to fungus mainly include immunomodulatory, antioxidant, anti-inflammatory, and antidiabetic effects (48). Our result showed the significant benefit of consuming fungus in improving the verbal memory scores. Although fungus is often seen in Chinese cuisine, the lack of studies exploring the role of fungus in the cognitive function indicates the need for further studies.

In our results the starch-rich dietary pattern, in particular salted vegetable and legumes, were positively associated with cognitive decline. Salted vegetables often called “pickled vegetables,” are an integral part of the diet in many families in China. It is often prepared by keeping tightly packed vegetable in a jar packing for a few weeks, allowing fermentation and growth of fungi and yeasts that can generate potentially carcinogenic N-nitroso compounds and mycotoxins (49). Studies showed the beneficial role of fresh brassica species (broccoli) in reducing the risk of cancers (50). However, there were positive links between pickled vegetable and increased risk of cancer, including esophageal (49) and gastric cancer (51), but studies of exploring the association between pickled vegetable and cognitive function were scarce. Pickled vegetable is often studied in a specific dietary pattern, which used to examine the association between that specific dietary pattern and cognitive function for older people. However, the study results were inconsistent. For example, a cross-sectional study in Japan showed that pickled vegetable was included in the “Plant food and fish” dietary pattern, which was significantly associated with high cognitive function (23); but our study indicated that the pickled vegetable was the key component which may the most influential diet factors for cognitive decline. Further research is needed, particularly to explore the mechanism of this association.

Legumes are often studied as part of a specific dietary pattern when assessing association between diet and cognition among older people, however, the research results were inconsistent. The results from a study conducted among participants who lived in the Mediterranean area showed the positive link between legumes pattern and cognitive function (52); but a 10 years follow-up study conducted in UK indicated that high legumes consumption included in the inflammatory dietary pattern was associated with high circulating serum interleukin-6 levels and accelerated cognitive decline at older ages (53), which was similar with our study results. Our results supported that compared with legumes non-consumers, legumes consumers had lower global cognitive scores and verbal memory scores.

The association between hypertension and cognitive function was consistent with previous studies. Moreover, cross-sectional and longitudinal studies implicate hypertension may be a possible contributor to late-life dementia. This aligns with cardiovascular disease as a major risk factor for stroke and dementia, and the strong association between hypertension and cardiovascular disease, thus far, hypertension is recognized as the most consistent risk factor for stroke and dementia (54). Due to the association between dietary pattern and hypertension (12), our hypothesis was that there may have been an interaction between hypertension and dietary pattern, with this interaction impacting on cognitive functioning. However, our results did not indicate this interaction, but does support the significant association between hypertension and cognitive functioning.

The strengths of the present study include using cumulative mean factor scores to capture the long-term diet pattern which may reduce dietary measurement error. In addition, the longitudinal study assists in making an etiological link between dietary pattern and cognitive function. However, some limitations may apply. The components in the global cognitive score of “orientation,” including asking participants current date and naming the tool were not asked in 2006. Therefore we were unable to use the entire global cognitive function score. The benefit of consuming milk and soy milk has been highlighted in the present study. However, due to the low amount of consumption and relative small proportion of people consuming milk and soy milk, it was difficult to draw the conclusion of the beneficial role of consuming milk and soy milk in maintaining cognitive function among older Chinese population. Lastly, the self-reported data of comorbidities (e.g., diabetes and hypertension) may have response bias in the study.

## Conclusion

We found a protein-rich dietary pattern was significantly negatively associated with cognitive decline, while starch-rich dietary pattern was positively associated with cognitive decline among a Chinese older population. Hypertension impacts on older people's cognitive functioning. The present study provides important evidence that can inform dietitians, other health professionals and educators to design dietary interventions for older people (such as encouraging older people to eat more protein-rich food, but less pickled food) aimed at improving and maintaining their cognitive function.

## Author contributions

XX and DP designed the study. XX conducted the analyses and wrote the manuscript. ZS guide the data analysis procedure. ZS, DP, JB, JH, and LH review the manuscript. All authors approved the final version of the manuscript.

### Conflict of interest statement

The authors declare that the research was conducted in the absence of any commercial or financial relationships that could be construed as a potential conflict of interest.
